# An Analysis of Biomechanical Parameters in OTP Police Physical Intervention Techniques for Occupational Risk Prevention

**DOI:** 10.3390/ijerph19116615

**Published:** 2022-05-28

**Authors:** José C. Vera-Jiménez, Felipe L. Meléndez-Sánchez, José A. Álvarez, Jesús Ayuso

**Affiliations:** 1 Municipal Police of Cadiz, Police Technology Area, Public Safety School of Council of Cadiz, 11010 Cadiz, Spain; 2 Department of Criminal Law and Criminology, Faculty of Law, National Distance Education University (UNED), Calle Obispo Trejo Number 2, 28040 Madrid, Spain; fmelendez@der.uned.es; 3 Department of Physical Chemistry, Faculty of Sciences, INBIO, University of Cadiz, 11510 Puerto Real, Spain; joseangel.alvarez@uca.es (J.A.Á.); jesus.ayuso@uca.es (J.A.)

**Keywords:** ergonomic, occupational risk prevention, police physical intervention, use of force, operational tactical procedure, motion capture technology

## Abstract

(1) Background: a set of ergonomic parameters that are relevant for risk assessment methods for the prevention of occupational risks, such as REBA or NIOSH, have been measured by means of inertial sensors that allow capturing the movements of the human body. These methods base their assessment on a number of postural and dynamic parameters. In the case of police physical intervention techniques, trunk, legs, arms, forearms and wrists angles, joint contact force and sheer force at the L5-Pelvic junction, asymmetry (angle and factor), and muscle power are the more relevant parameters to be considered. (2) Method: The data have been collected by means of a motion capture suit equipped with 19 inertial sensors. The large amount of data and the 3-dimensional plots have been managed by a powerful software package specific for ergonomic analysis. The police physical intervention technique used was OTP. (3) Results: Five ergonomic parameters in a traditional police physical intervention technique have been analyzed. REBA scores and ergonomic metrics have been recorded and discussed with some prevention risk limits from the literature. (4) Conclusions: the usage of inertial sensors to capture the movements in OTPs provides a new and quite an efficient viewpoint for occupational risk research studies.

## 1. Introduction

In recent times, the use of inertial sensor-based methodology has increased in medical applications [1]. Thus, for example, inertial measurement units (IMUs) that analyze human motion using a wearable sensor platform [2,3] have become widely used tools for the rehabilitation or prevention of injuries.

An extensive bibliography can also be found on biomechanical studies of different sports based on IMUs. For instance, the compilation carried out by Van der Kruk et al. [4] is worth mentioning because they carried out an in-depth analysis of the precision of measurements based on IMUs for their application in fast-moving activities, such as sports activities. However, not many of these studies focus on police or security forces’ physical interventions, which is the scope of the present research.

Among the technical publications found by us on police interventions, there is a study by Mavor [5] on the effects of load carriage on military or defense forces based on optical motion and Inertial Measurement Unit sensors (IMU), as captured when performing military-like movements. In it, several parameters such as full-body joint angles were determined through movements such as running, walking, kneeling, and prone positioning. Based on that data, the IMU system proved to be suitable to capture and to reconstruct full-body movements and their variability in military activities. However, the movements covered were rather simple, and a more detailed analysis of a wider range of physical intervention techniques would be necessary—e.g., upper body movements.

A biometrical analysis of the most important movements performed during police physical interventions would be of great interest for the development of risk prevention studies.

The present paper intends to set the basis for reducing occupational risks associated with police physical intervention techniques. It will be particularly focused on an operational tactical procedure (OTP) [6] that consists of the control of an opponent by a police officer according to the methods and the techniques taught in police training academies. OTPs are different from traditional procedures, which were based on martial arts and combat sports, and whose purpose was to immobilize an opponent. Those types of interventions involved a high risk of injury to both the adversary and the police officer. On the contrary, OTPs avoid hitting any vital areas and causing serious damage to the opponent.

Our study approaches prevention based on ergonomic evaluations. For this reason, certain types of biometric parameters such as frontal or sagittal plane angles at the knee, hip, or spine must be accurately determined for a correct evaluation. In this sense, IMU-based 3D motion capture tools have demonstrated their accuracy level when it comes to determining these parameters [7].

A Rokoko 19-IMUs Suit has proven to be a reliable, cross-platform (iOS, MS Windows, etc) option for this purpose. In addition, the Biomechanics of Body (BoB) modeling software package developed by Shippen [8] has been used.

This hardware and software combination has proven to be a powerful piece of equipment for the analysis of human biomechanics ergonomic factors, so they have been used to calculate joints’ motion ranges, their trajectories, joint torques, muscle forces, ground reaction forces, and joint contact forces.

A number of different ergonomic evaluation methods such as REBA [9,10], NIOSH [11], RULA [12], OWAS [13], LEST [14], JSI [15], NMQ-E [16], Snook and Ciriello Tables [17], OCRA [18], OCRA Checklist [19], and the Chaffin Biomechanical Model [20] have been developed. Of them all, this study will take into consideration the main characteristics and parameters of the REBA method–as a method that evaluates postural loads–and the NIOSH method, which is intended for the evaluation of weight handling. These two methods will be the basis for our analysis of the ergonomics in police physical intervention techniques (PIT) for an operational tactical procedure or OTP (Vera-Jiménez et al., 2020) [6].

It could be argued that as ergonomic evaluation methods, REBA and NIOSH are not the most adequate methods for the evaluation of physical intervention techniques. Nevertheless, despite their undeniable deficiencies, some of the parameters they evaluate can be rather useful and particularly interesting for the purposes of this study.

The National Institute for Safety, Health, and Wellbeing at Work, under the Ministry of Labor and Social Economy of Spain, has collected REBA and NIOSH methods as Tech-nical Notes of Prevention (NTP is its Spanish acronym). These NTPs are guidelines and recommendations for good practices and, unless they are included in the regulations in force, they are not mandatory. When considering the suitability of the recommendations contained in a particular NTP, its publication date should be taken into account.

### 1.1. Parameters of Interest in the REBA (Rapid Entire Body Assessment) Method

Designed by Nogareda [10] and included as NTP 601, the REBA method intends to evaluate a wide set of parameters related to human anatomical geometry, such as the angles and the relative positions of the limbs and other parts of the body, according to which a series of scores are assigned based on specific intervals and limit values of these geometric parameters.

By applying this method, the postures of the upper extremities, lower extremities, trunk, and neck, while making a difference between the right and left side of the body, are evaluated. Thus, each part of the body is assigned a score that will be higher as its position is further away from its most relaxed posture. The REBA method also takes into account the type or form of the grip, and it will score higher the more forced the grip.

The maximum total REBA score is 15, and it comprises five value ranges. A risk index and the corresponding recommendations or interventions are established according to the different risk levels. The risk index can go from negligible to very high risk as follows: negligible risk, with no action recommended; low risk, with some changes to be considered; medium risk, where some changes are needed; high risk, with changes required as soon as possible; and very high risks, where changes are to be implemented immediately.

In the case of police physical intervention techniques, trunk (Table 1), legs (Table 2), arms (Table 3), forearms (Table 4), and wrists angles (Table 5) are the more relevant parameters to be considered as depicted in the figures below.

The scores will be increased by 1 in the following cases (Table 6):

### 1.2. Parameters of Interest in the NIOSH Method

This study will focus mainly on the risks associated to low back pain or back problems; it has been considered that the method based on the NIOSH lifting equation [11] would be the most appropriate methodology to be used. Therefore, based on the NIOSH equation, we have established the compression force limit at the L5-Pelvic junction, measured in Newtons (N) and the angle of asymmetry (A).

#### Asymmetry Angle and Asymmetry Factor

The asymmetry angle (A) is an indicator of the torsion/bending of a worker’s trunk during a lifting or loading task. Therefore, it indicates the non-symmetrical movements with respect to the sagittal plane of the human body.

**Figure 1:**
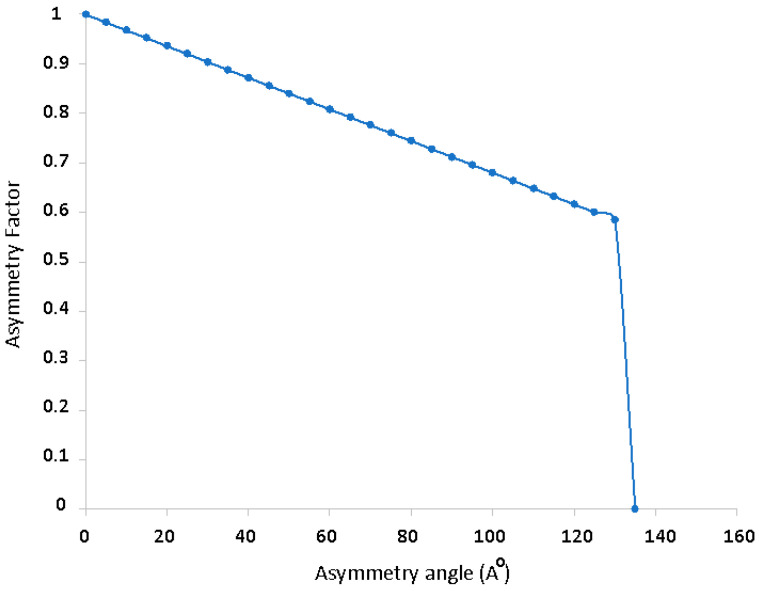
Lifting weight percentage recommended by the NIOSH method depending on the asymmetry angle.

**Figure 2:**
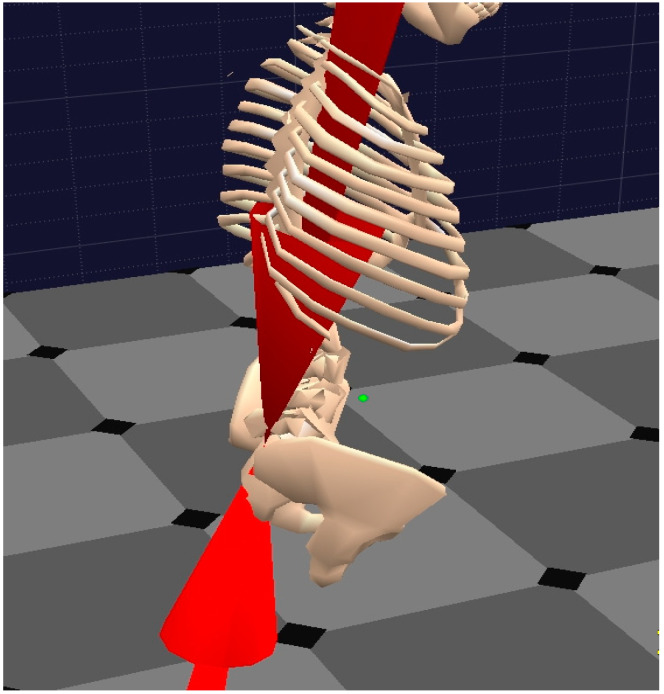
Pinch force 3D vector representing the compression force at the L5-Pelvis junction.

According to NIOSH, lifts that require torsion of the trunk are penalized, that is, asymmetrical lifts should be avoided. To do this, the asymmetry factor (AM) is calculated using the formula: AM = 1 − (0.0032 ∗ A), where A is the rotation angle (in sexagesimal degrees) as shown in Figure 1 and Figure 2.

The AM factor takes the value 1 when there is no asymmetry, and its value decreases as this angle increases. It will also be considered that when A > 135°, AM will be given the value 0 (Figure 1).

The latter is the worst-case scenario, where no weight lifting would be recommended (indicated by RWL = 0).

In the most extreme cases, people affected by sagittal imbalance usually present a significant alteration of their gait pattern, their ranges of joint mobility, and the force and muscle work capacity in their lower limbs [21].

Sagittal imbalance is significantly correlated with a decrease in the activation force of all the major muscle groups in the lower extremities (gluteus medius, psoas, hamstrings, etc.) with the exception of the quadriceps.

In addition, the research of González-Míguez [21] makes the assessment that by means of three-dimensional movement analysis, with a system such as that used in the present study, the usual underestimation detected by conventional radiographic diagnosis could be avoided.

### 1.3. Joint Contact Force at the L5-Pelvic Junction

The most relevant compression forces to be considered are those that act on the spinal discs, including body weight and load behavior. According to the criteria of the NIOSH [11], 3400 N at the L5-Pelvic junction (Figure 2) has been established as the compression force limit for risk of low back pain to appear. 

### 1.4. Sheer Force at the L5-Pelvic Junction

**Figure 3:**
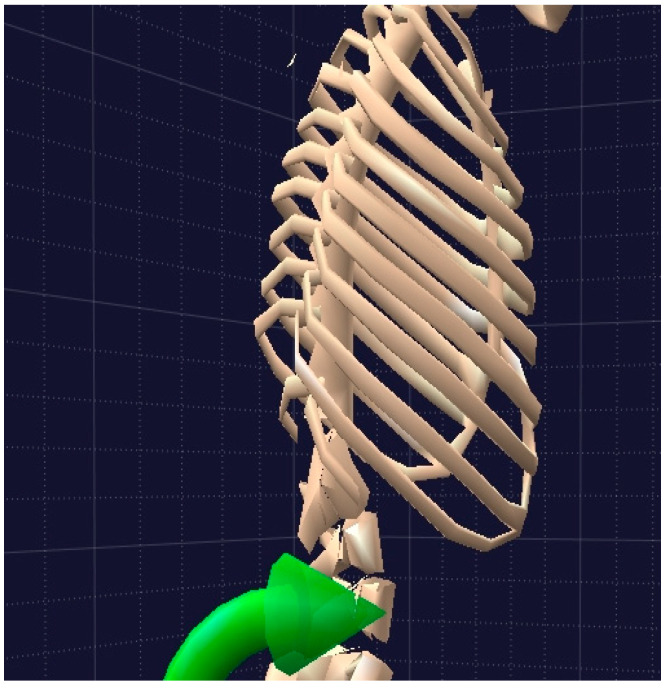
Sheer force at the L5-Pelvic junction represented by a 3D vector.

Sheer force is that which causes a parallel sliding or displacement of a vertebra with respect to the immediately lower or upper one. Therefore, it is force that is perpendicular to that of compression. Sheer force typically arises when the trunk of a human body makes pulling and/or pushing efforts (Figure 3).

According to an extensive biodynamic engineering study [22] on the most common causes of low back pain, the usual range of these forces is between 600 and 3200 N, with an average value of 1700 N. This paper concludes that sheer force up to 1000 N in low frequency tasks (≤100 loads/day) would be acceptable for 90% of the working-age population, while sheer force of 700 N would be tolerable in higher-frequency tasks (around 1000 loads/day).

Obviously, all of these dynamic parameters will strongly depend on the mass, height, age, and gender of the police officer performing each physical intervention technique.

### 1.5. Total Muscle Power

Muscle power is another dynamic parameter that has been considered of interest for the present study. The same biomechanical calculation software, Bob, was used to retrieve the corresponding data. Muscle power calculations are based on the amount of energy used to complete a specific work in a given time, and it is expressed as watts.

This parameter has often been analyzed in sport training for many years. Specially for certain sports, such as tennis, baseball, or golf [23,24,25,26]. However, it is also the object of study in certain jobs where muscle power is associated to a series of typical pathologies. In this sense, the Instituto Nazionale per L’Assicurazione contro gli infortuni sul lavoro [26] carried out a study on the prevalence of musculoskeletal disorders (MSD) suffered by EU workers from the construction sector as a result of performing tasks that put workers at risk, such as the manual lifting of loads, forced postures, etc.

In this study, the most commonly detected injuries include ulnar collateral ligament (UCL) tears, flexor–pronator tendinosis or tears, ulnar neuritis, posterior impingement, ostechondritis dissecante capitellum and extensor tendinopathy, i.e., tendinopathies are the most common injuries.

One of the main purposes of this study is to provide an overview of the injuries of the upper extremities (shoulders, elbows, and wrists) and their prevention based on the current knowledge of biomechanics since we agree with Pluim [24] that the biomechanical analysis of the muscular power, forces, loads and muscle movements should lead to a better understanding of the pathophysiology of the injuries.

## 2. Materials and Methods

### 2.1. The Biomechanics of Bodies (BoB) Software

**Figure 4:**
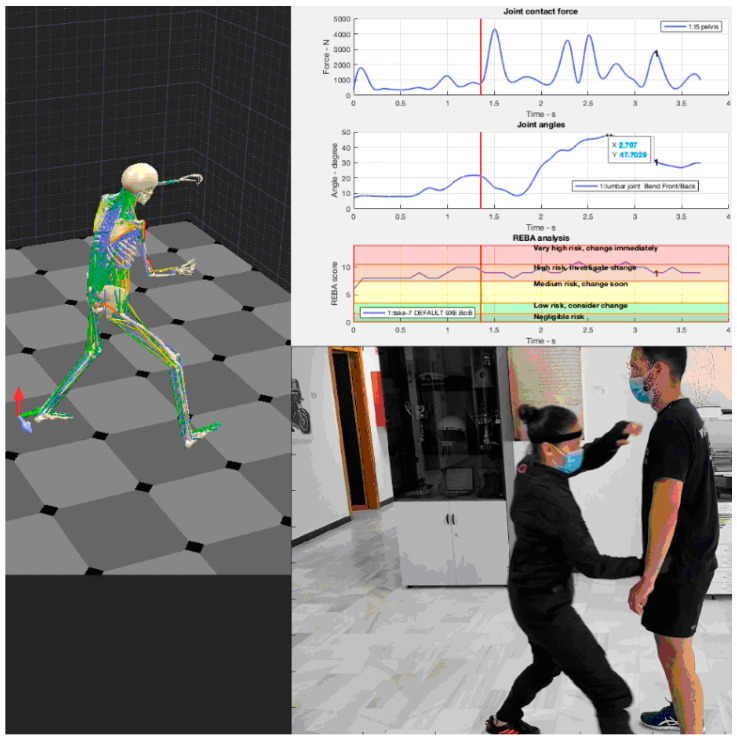
Graphics generated by means of BoB software package.

This is a family of biomechanical modeling software packages that uses the data corresponding to positions, speeds, and accelerations, provided by the sensors to determine linear and angular speeds and accelerations (from muscular extensions or rotations) as well as other dynamic measures, such as muscle tension/compression forces, energy, and power exerted by them. All of these data are valuable for the purposes of this study. BoB also provides three-dimensional graphics and a user interface where the results from the analysis can be displayed (Figure 4).

This software provides a number of options to register the values of interest for the REBA analysis. Thus, for grip quality, the following four options are available: good fitting handle and mid-range power grip (this is the most appropriate for OTPs); macceptable but not ideal handhold; not acceptable but possible handhold; no handhold, awkward or unsafe. It also provides four options on “Degree of activity?” as follows: one or more body parts held still for over 1 min; more than 4 repeated small range actions per minute; actions causing rapid change in posture (this is the most appropriate for OTPs); none of the above.

### 2.2. Rokoko Smartsuit Pro

**Figure 5:**
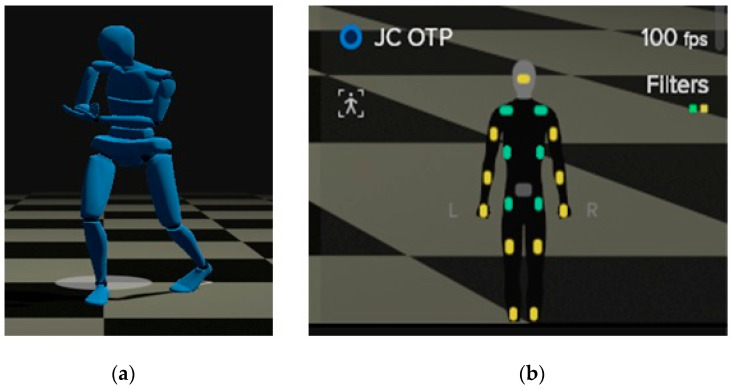
(**a**) Avatar illustrating the posture of a human body while performing an intervention technique. (**b**) Arrangement of the wireless sensors on a human body.

This piece of equipment consists in a suit fitted with 19 active (triaxial accelerometric, gyroscopic, and geomagnetic) wireless inertial sensors that allow the determination of the position, speed, acceleration, and magnetic fields in a human body. It also features a specific software application that displays on screen the body positions as avatars (Figure 5). The sensors can record data up to 100 times per second.

### 2.3. OTP Police Physical Intervention Techniques

Police physical intervention techniques are based on operational tactical procedures (OTP), which comprise a series of physical intervention techniques (PIT) that use defense mechanisms such as blocking, diverting, and grabbing of the upper and the lower limbs in order to avoid hitting vulnerable and vital areas to minimize the risk of severe damage.

In fact, the techniques described in the OTP procedures rely on inflicting controlled pain, for example hitting the triceps tendon or quadriceps muscle can be painful enough to completely block and control the opponent. These procedures avoid serious damage not only to the opponent but also to the police officer [6].

### 2.4. Research Collaborators

The values of the measurements of all the above biomechanical parameters were recorded when the entire process of a classic physical intervention technique was carried out with two expert male police officers wearing the sensorized suits.

The physical characteristics are female 1.65 m tall and 62 kg in weight for the officer who served as the police, and male 1.80 m tall and 80 kg for the officer who served as the opponent.

## 3. Results and Discussion

A large set of biometric parameters have been simultaneously determined thanks to the biomechanical analysis performed by means of this new capturing technology based on wireless sensors. This ergonomic study based on human factors should allow us to determine the probability of injury.

**Figure 6:**
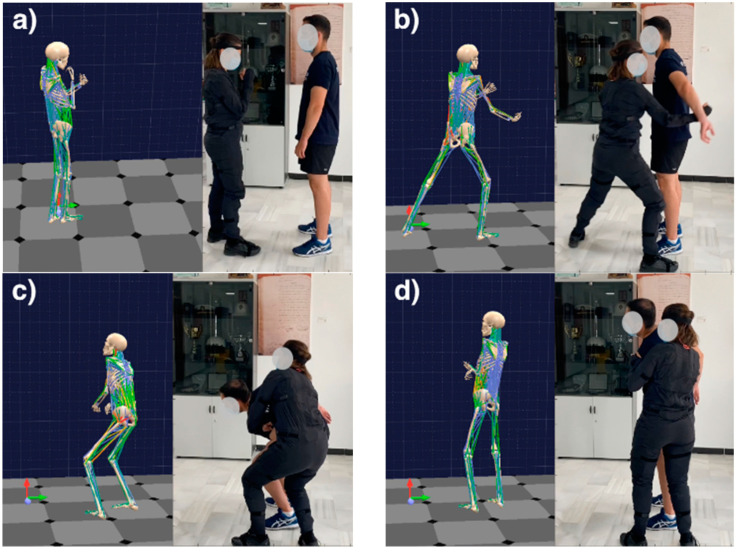
Snapshots of four steps in an OTP implementation: (**a**) at 0.0 s, (**b**) at 1.5 s, (**c**) at 2.6 s, and (**d**) at 3.6 s. Left: Anatomical avatar corresponding to the posture of the police officer on the right hand side image.

The software application allows the use of avatars that correspond to the position of the human body at specific moments in a particular OTP. Figure 6 shows four moments in an OTP performed by a female police officer facing an opponent. The officer is applying an immobilization technique to a citizen.

Image Figure 6a, shows the initial moment of the process, that is, the safety position against an aggressor. The image on the left shows the anatomical avatar corresponding to the police officer’s posture.

Image Figure 6b in the same figure shows the police officer stepping forward, using the leg on the side that is closest to the spot where she is moving and at the same time, she uses her forearm (from the same side) to reach for the opponent’s elbow.

In the image Figure 6c, the opponent is dragged down to the officer’s hip, which is a step prior to his immobilization. Finally, image Figure 6d shows how the opponent is immobilized in a vertical position, where he should be perfectly under control.

Throughout the whole OTP implementation, the sensors are recording biomechanical parameters that are then transformed into a set of useful scores used by REBA or NIOSH to evaluate the situation and accordingly prevent any possible occupational risks.

### 3.1. Assessment REBA

**Figure 7:**
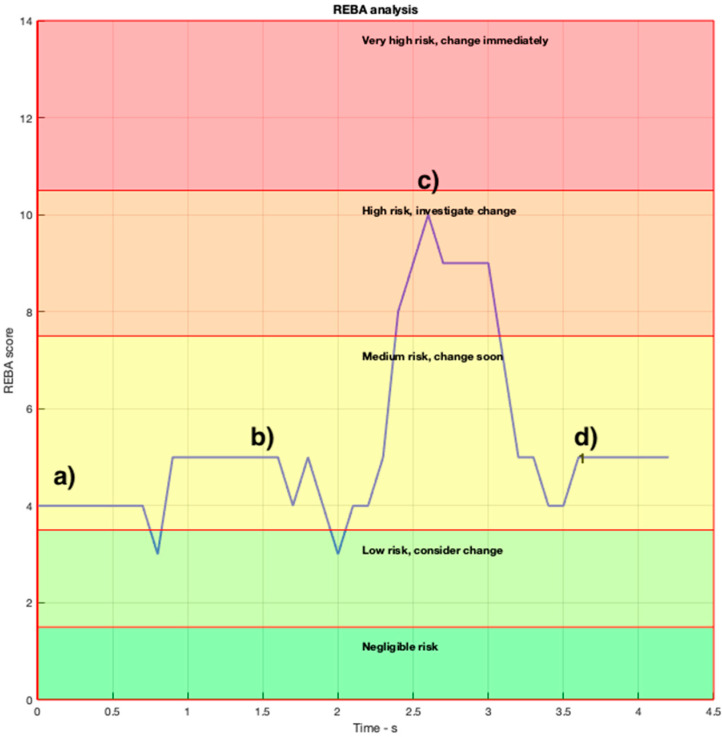
REBA scores of the OTP. The instants corresponding to the four snapshots in Figure 6 are indicated from (**a**–**d**).

Figure 7 shows the evolution of the REBA scores throughout the OTP implemented for this study. The REBA values are registered 100 times per second, and the 4 snapshots in Figure 6 are labeled (a) to (d). As can be seen in this figure, the scores of the OTP selected for the present study do not reach the level “Very high risk”, while its average score is within the “Medium risk” range. This is an unusual score that corresponds exclusively to this particular case.

### 3.2. Asymmetry

**Figure 8:**
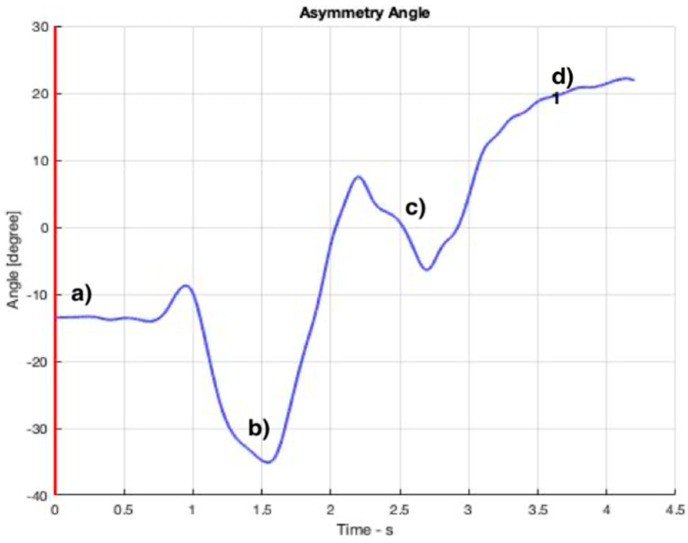
Value of the asymmetry angle throughout the OTP. The instants corresponding to the four snapshots in Figure 6 are indicated from (**a**–**d**).

**Figure 9:**
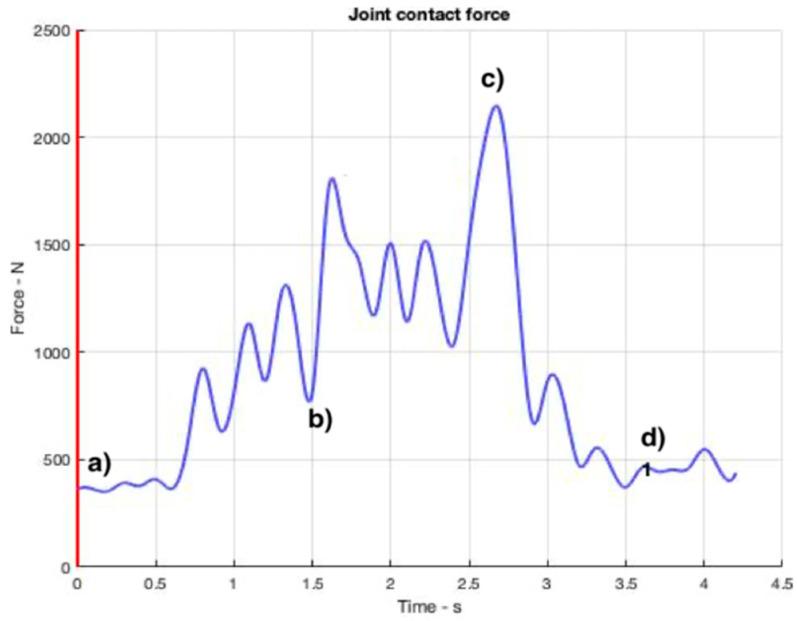
Joint contact force values for compression at the L5-Pelvic throughout the OTP. The instants corresponding to the four snapshots in Figure 6 are indicated from (**a**–**d**).

**Figure 10:**
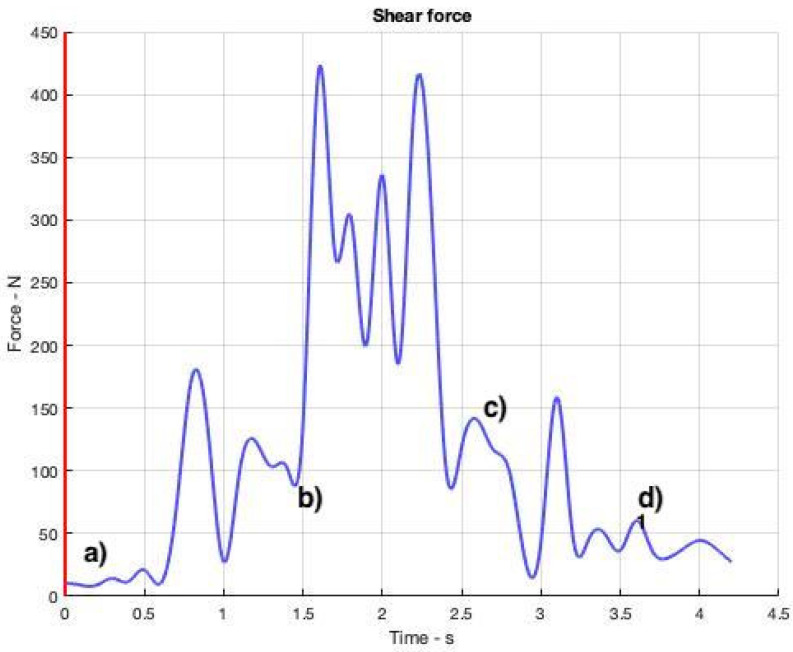
Sheer force values at the L5-Pelvis joint throughout the OTP. The instants corresponding to the four snapshots in Figure 6 are indicated from (**a**–**d**).

**Figure 11:**
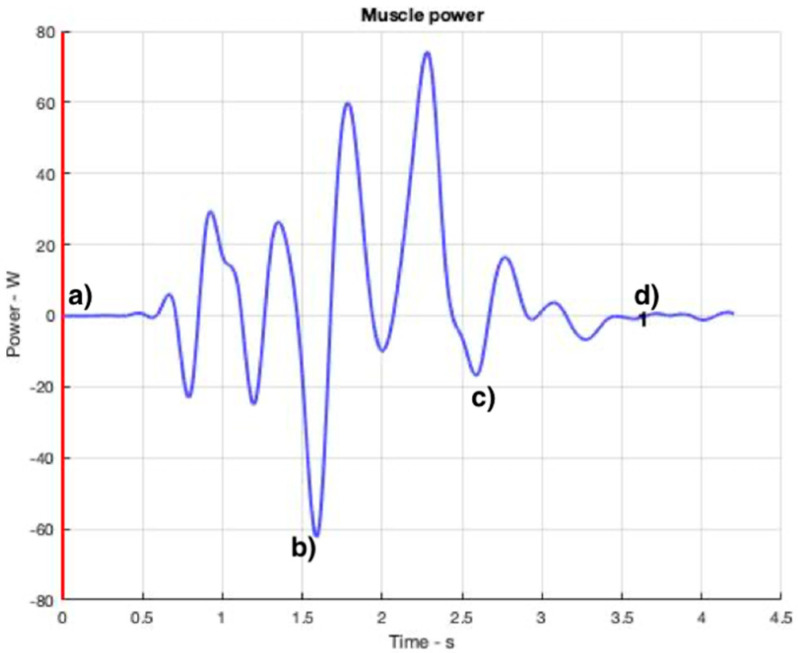
Total muscle power throughout the OTP. The instants corresponding to the four snapshots in Figure 6 are indicated from (**a**–**d**).

Figure 8, Figure 9, Figure 10 and Figure 11 show curves in blue that correspond to asymmetry angle and factor), joint contact force, sheer force at the L5-Pelvic junction, and muscle power, respectively.

The variation of the asymmetry angle (Figure 8) ranges between −35° and 25° degrees, the torsion starts from the first second of the intervention and the asymmetry factor decreases no more than 15% of the lifting weight.

### 3.3. Joint Contact Force

The joint contact force, or compression force at the L5-Pelvis junction changes very fast, as can be seen from Figure 9. Its maximum value (2146 N) is reached at the moment when the police officer forces her opponent to bend down (Figure 9c).

One of the parameters of particular interest in this paper is the sheer force on the segment between the L5 vertebra and the pelvis since in a previous study [8] it had been reported that clear differences between expert and novice workers could be observed, an aspect that is worthy of note from a risk prevention point of view.

Sheer force often occurs due to the force of gravity acting on the upper body when bending the trunk forward, but it can also be quite significant in some jobs involving the pushing or pulling of objects [27]. Sheer force is generally defined as force that acts parallel to the plane determined by the intervertebral discs within the specific segment of interest [28].

### 3.4. Sheer Force

It can be seen in Figure 10 that the sheer force at the L5-Pelvic joint is moderate (not so high) throughout the transition until the officer steps forward. Then, it increases from Figure 10b to Figure 10c, when the police officer leans forward toward her opponent. The maximum value (418 N) reached by the sheer force in this example takes place at 1.6 s.

### 3.5. Muscle Power

Another very important parameter from the point of view of occupational risks is the muscular power exerted by the police officer. Figure 11 represents the total muscular power as positive and negative values, where positive values are assigned to eccentric movements (that is, the muscle force is exerted in the opposite direction of muscle movement). Conversely, concentric motions are related to the negative values. If power absolute values (rate of energy consumed per unit of time) are considered, the highest muscle power consumption takes place between steps Figure 10c and Figure 11b.

## 4. Conclusions

The application of Inertial Measurement Unit (IMU) sensors to capture movements represents a new and a highly efficient approach for studies on the occupational risks of OTP.

Through this study, IMU-based 3D motion capture tools have demonstrated their suitability for the measuring of the biometric parameters that are used in REBA and NISOH assessment methods, such as trunk frontal and sagittal plane angles; arm, forearm, wrist, and leg angles, etc. Furthermore, other dynamic parameters, such as joint contact force and sheer force at the L5-Pelvic junction, asymmetry (angle and factor), and muscle power have also been determined.

IMU technology is also easy to use outside laboratory environments.

All together, we have concluded that these are suitable tools that can be used to determine the risk level of other physical intervention techniques.

## 5. Patents

Vera-Jiménez, J.C.; 2017; Versatile protector suitable for carrying defenses for police use and other accessories, and for the use of defensive blocking techniques. Methods of employment (Machine-translation by Google Translate, not legally binding) ES2615602A1 (B2) • 7 June 2017.

## Figures and Tables

**Table 1:** Trunk angles.

	Score:
Upright position (Vertical angle close to 0°)	1
Slightly bent position (−20° to 20° angle from the vertical line)	2
Bent–flexion (20° to 60° forward angle) or extension (−20° backward angle)	3
Very inclined position (flexion over 60°)	4
When accompanied by twisting or lateral flexion of the trunk, the score increases by one unit.	+1

**Table 2:** Leg angles.

	Score:
Bilateral weight bearing, walking, or sitting	1
Between 30° and 60° knee flexion	+1
Unilateral weight bearing, light support, or unstable posture	2
Over 60° knee flexion	+2

**Table 3:** Arm angles.

	Score:
Between 0° and 20° flexion/extension from the vertical line	1
Between 21° and 45° flexion or >20° extension from the vertical line	2
Between 46° and 90° flexion from the vertical line	3
Over 90° flexion from the vertical line	4
When accompanied by the abduction or the rotation of the shoulder	+1
When accompanied by the raising of the shoulder	+1
When the posture is gravity assisted	−1

**Table 4:** Forearm angles.

	Score:
60° to 100° flexion/extension angle	1
<60° or >100° flexion (bending) from the vertical line	2

**Table 5:** Wrist angles.

	Score:
Between 0° and 15° flexion/extension from the horizontal line	1
Over 15° flexion/extension from the horizontal line	2
When there is also twisting or lateral deviation	+1

**Table 6:** Score Increase.

	Increase:
One or more body parts remain static for a time, e.g., posture held for more than 1 min.	+1
Repeated short movements, e.g., repeated more than 4 times/minute.	+1
Rapid and major postural changes or unstable postures	+1

## Data Availability

Not applicable.

## Abbreviations

BoB	Biomechanics of Body
IMU	Inertial Measurement Units
JSI	Job Strain Index
LEST	Laboratoire d’Economie et Sociologie du Travail (Labor Economics and Sociology Laboratory)
NIOSH	National Institute for Occupational Safety and Health
NMQ-E	Nordic Musculoskeletal Questionnaire (Extended)
OCRA	OCcupational Repetitive Action
OTP	operational tactical procedure
OWAS	Ovako Working Analysis System
PIT	Physical Intervention Techniques
REBA	Rapid Entire Body Assessment
RULA	Rappid Upper Limb Disorders
